# Possible Improvement of Tardive Dyskinesia and Negative Symptoms With Lurasidone Augmentation in a Treatment‐Resistant Schizophrenia Patient

**DOI:** 10.1155/crps/7121649

**Published:** 2026-08-11

**Authors:** Umut Günay, Candost Demiröz, Fatma Seher Kocaayan, İlay Dalkıran, Elif Tatlıdil

**Affiliations:** ^1^ Department of Psychiatry, Kocaeli University, İzmit, Kocaeli, Türkiye, kocaeli.edu.tr

**Keywords:** case report, lurasidone, negative symptoms, tardive dyskinesia

## Abstract

Tardive dyskinesia (TD) is a cluster of symptoms affecting orofacial, truncal, and extremity regions following prolonged exposure to antidopaminergic agents and may be life‐threatening in severe cases. It is most commonly observed in patients receiving long‐term antipsychotic treatment, such as individuals with schizophrenia. Lurasidone is a relatively novel antipsychotic that has been reported to induce tardive syndromes in patients with schizophrenia and bipolar disorder and has also been used as an augmentation strategy in treatment‐resistant schizophrenia. In this report, we present an intriguing case in which TD improved following the addition of lurasidone to the patient’s existing treatment, accompanied by a reduction in negative psychotic symptoms. The patient’s Abnormal Involuntary Movement Scale (AIMS) score decreased from 20 to 8–9 after the initiation and subsequent dose increase of lurasidone. Additionally, the Positive and Negative Syndrome Scale (PANSS) total score decreased from 171 to 148, with particularly notable improvement in the negative symptom subscale. The beneficial effect of lurasidone on TD is hypothesized to be related to various receptor interactions of the current treatment, as well as 5‐HT_2_A antagonism and preferential modulation of dopamine turnover in the prefrontal cortex rather than the striatum, as also observed with clozapine. Further studies are warranted to elucidate the underlying mechanisms of this phenomenon.

## 1. Introduction

Tardive dyskinesia (TD) is a syndrome that manifests as choreoathetoid movements of the lips, mouth, and tongue and, in severe cases, involves the trunk and extremities. It occurs following exposure to antidopaminergic medications and is typically expected to develop after at least 3 months of antidopaminergic medication use (1 month in elderly) and to persist for a minimum of 4 weeks after discontinuation of the offending agent. The risk of TD is higher among users of first‐generation antipsychotics; however, second‐generation antipsychotics also carry a relatively lower but clinically significant risk [1]. Among second‐generation antipsychotics, agents such as risperidone and paliperidone are thought to be associated with a relatively higher risk of TD; however, some studies suggest that there may be no significant difference in relative risk. Clozapine appears to be an exception, as it has been associated with a comparatively lower risk of TD [2, 3]. Identified risk factors for TD include female sex, the presence of mood symptoms, cumulative antipsychotic dose, duration of antipsychotic treatment, advanced age, having organic brain injury, and having diabetes mellitus [4].

Current treatment options for TD primarily include vesicular monoamine transporter (VMAT) inhibitors, and a recent meta‐analysis has also suggested that vitamin E may contribute to symptom improvement [5]. In the literature, olanzapine and quetiapine have also been reported to improve TD symptoms induced by both first‐ and second‐generation antipsychotics [6, 7].

Lurasidone is a relatively novel antipsychotic used in the treatment of schizophrenia and mood disorders. It binds strongly to dopamine D_2_, serotonin 5‐HT_2_A, and 5‐HT_7_ receptors; it is a partial agonist at 5‐HT_1_A receptors and shows moderate affinity for α_2_C, D_3_, α_2_A, and 5‐HT_2_C receptors. Its antihistaminergic and antimuscarinic effects are negligible [8]. Lurasidone’s potent 5‐HT_7_ binding has been hypothesized to account for its procognitive effects in schizophrenia, a domain in which many antipsychotics struggle to demonstrate improvement. Additionally, partial agonism at 5‐HT_1_A receptors may account for its antidepressant and anxiolytic effects. As with other second‐generation antipsychotics, 5‐HT_2_A antagonism could explain its lower propensity to induce extrapyramidal symptoms and may contribute to improvements in negative symptoms [9]. In the literature, several reports have suggested that lurasidone may be associated with the development of tardive syndromes such as orofacial TD, tardive dystonia, tardive akathisia, and truncal TD [10–13]. In this case report, we present an interesting observation of improvement in both TD and psychotic symptoms following the addition of lurasidone to the treatment regimen of a patient with treatment‐resistant schizophrenia.

## 2. Case

A 40‐year‐old, single, male patient had been first admitted to the Kocaeli University Psychiatry Outpatient Unit in 2005 with prominent psychiatric symptoms, including visual and auditory hallucinations, persecutory delusions, Schneiderian first‐rank symptoms, magical thinking, nihilistic delusions, and disorganized behavior. He had been diagnosed with paranoid schizophrenia. Throughout the course of his illness, multiple antipsychotic medications were trialed, either as monotherapy or in combination. These included risperidone 4 mg/day, zuclopenthixol decanoate 200 mg weekly, zuclopenthixol decanoate 200 mg weekly combined with olanzapine 10 mg/day, paliperidone palmitate 150 mg/month, and risperidone long‐acting injection 50 mg biweekly. Despite these treatments, his positive and negative symptoms largely persisted, and he experienced multiple inpatient admissions over the course of the illness. After an inadequate response to two different antipsychotic trials, a diagnosis of treatment‐resistant schizophrenia was made. However, due to poor treatment adherence and patient’s delusional belief toward healthcare staff, clozapine therapy could not be initiated. During his last inpatient admission in 2017, zuclopenthixol decanoate 200 mg and olanzapine 20 mg/day were administered. No further inpatient admissions occurred until 2021; however, due to persistent negative symptoms, fluoxetine 20 mg/day was added to his treatment regimen. During the course of treatment, parkinsonism and tremor emerged, leading to the addition of biperiden 6 mg/day and propranolol 20 mg/day, respectively. In 2021, the patient had developed involuntary lip and mouth movements with a choreoathetoid pattern. Neurological evaluation, including neuroimaging, revealed no structural pathology. Over the following month, these involuntary movements had worsened and had extended to the trunk. Symptoms had been alleviated by voluntary movement and had been absent during sleep. The patient had been evaluated using the Abnormal Involuntary Movement Scale (AIMS), with a total score of 25, indicating severe TD. Zuclopenthixol decanoate was gradually discontinued, and olanzapine was increased to 30 mg/day. Due to adherence issues and insurance limitations, VMAT2 inhibitors could not be initiated. Exacerbated psychotic symptoms had warranted the initiation of amisulpride 400 mg/day. Although TD symptoms remained severe (AIMS score ~20), no further progression had been observed. Amisulpride had been subsequently increased to 1200 mg/day.

In 2025, the patient was reevaluated. He continued to experience prominent Schneiderian and persecutory delusions, lacked insight, exhibited impaired rational thinking, and was unable to work due to persistent positive and negative symptoms. His Positive and Negative Syndrome Scale (PANSS) score was 171. An electrocardiogram (ECG) showed a normal QTc interval, and lurasidone 20 mg/day was added to the treatment regimen. One week after initiation of lurasidone, the patient began engaging in self‐care activities and demonstrated increased motivation to return to work. Lurasidone was increased to 40 mg/day. Within 1 week of lurasidone dose increase, a marked improvement in choreoathetoid movements was observed, beginning in the upper and lower extremities and trunk. Subsequently, the patient reported showering twice weekly, using public transportation independently, and maintaining occupational functioning without psychotic exacerbation. His PANSS score decreased to 148. Finally, involuntary movements of the lips, tongue, and jaw diminished substantially and nearly resolved. The AIMS score decreased to 8–9, indicating a significant clinical improvement in TD. Lurasidone dose was increased to 60 mg/day in the following week and to 80 mg/day in the subsequent week. Meanwhile, the doses of amisulpride and biperiden were gradually reduced. The final treatment was lurasidone 80 mg/day, olanzapine 30 mg/day, fluoxetine 20 mg/day, and propranolol 20 mg/day. During the follow‐up period, there was no exacerbation of TD or psychotic symptoms. AIMS and PANSS assessments were conducted by a single rater in accordance with the respective scale manuals. A detailed clinical history of the patient is presented in Table 1. Also, changes in PANSS and AIMS scores along with clinical work‐up of the patient are presented in Table 2. During the evaluation, the differential diagnosis of other movement disorders was also considered. Differential diagnoses and the reasons for their exclusion are presented in Table 3.

**Table 1:** Longitudinal clinical course, treatment interventions, and outcomes of the patient.

Year	Occasion	Treatment and dosage (mg/day, mg/week, mg/month)	Outcome	
2005	The patient had presented to the psychiatry outpatient unit for the first time with prominent psychiatric symptoms, including visual and auditory hallucinations, persecutory delusions, Schneiderian first rank symptoms, magical thinking, nihilistic delusions, and disorganized behavior. A diagnosis of paranoid schizophrenia had been made	Risperidone had been initiated and titrated to 3 mg/day, at which point a partial response had been observed. The dose had subsequently been increased to 4 mg/day		Marked functional impairment had persisted, while only partial alleviation of positive symptoms had been observed

2005	Due to marked functional impairment and persistent debilitating delusions, a change in treatment had been decided	A switch to zuclopenthixol decanoate 200 mg weekly had been made		Positive and disorganized symptoms had decreased slightly; however, negative symptoms and functional impairment had persisted

2005	After switching to weekly zuclopenthixol decanoate, parkinsonian symptoms had emerged, including bradykinesia and cogwheel rigidity	Biperiden had been added, and it had been titrated up to 4 mg/day to achieve symptom relief		Parkinsonian symptoms had improved

2005–2010	The patient had been followed up at the outpatient clinic; however, he had continued to exhibit marked negative symptoms and functional impairment, including difficulty maintaining personal hygiene (e.g., inability to shower adequately), impaired occupational functioning, and reduced ability to engage in conversation	Maintenance treatment with zuclopenthixol decanoate 200 mg weekly and biperiden 4 mg/day had been continued		Despite these limitations, his employers had decided to retain him in his job by assigning less complex tasks, and he had continued working

2010	A marked exacerbation of positive symptoms had occurred, including persecutory delusions that his colleagues would harm him. Concurrently, he had begun planning to harm his colleagues. Mandatory inpatient admission had been arranged, and the patient had been hospitalized. During the hospitalization period, olanzapine monotherapy had initially been administered and titrated up to 30 mg/day; however, no clinical improvement had been observed. A diagnosis of treatment‐resistant schizophrenia had been made. Clozapine had been considered; however, the patient had developed persecutory delusions toward healthcare workers and had refused to attend the clinic regularly for blood monitoring. In addition, he had reported that he could rarely leave his home due to his belief that his colleagues would harm him while going to work	At discharge, the final treatment regimen had consisted of zuclopenthixol decanoate 200 mg weekly, olanzapine 10 mg/day, and biperiden 4 mg/day		Homicidal thoughts had remitted; however, persistent delusions, negative and disorganized symptoms, and marked functional impairment had remained. He had stopped going to work; however, with encouragement from his employers, he had agreed to return and had been able to stay at work for only 1–2 h

2016	During an outpatient visit, the patient had exhibited marked command hallucinations and recurrent homicidal ideation toward his colleagues. Inpatient admission had been arranged	At discharge, the patient had been prescribed risperidone long‐acting injection 50 mg biweekly, oral risperidone 4 mg/day, and valproate 1000 mg/day		Although homicidal thoughts had remitted and positive symptoms had partially improved, negative symptoms had persisted, including poor self‐care (e.g., showering once weekly) and reduced spontaneous speech. Functional impairment had remained unchanged, as the patient had rarely attended work

2017	Given the persistence and further worsening of functional impairment—including neglect of financial responsibilities, avoidance of usual commuting routes due to delusion(s) and poor hygiene—a switch from risperidone long‐acting injection 50 mg biweekly to paliperidone palmitate 150 mg monthly had been planned	His treatment had been organized as paliperidone palmitate 150 mg monthly, olanzapine 10 mg/day, valproate 1000 mg/day, and biperiden 6 mg/day		Although there had been mild improvement in functioning, negative symptoms had slightly worsened, and mild depressive symptoms had emerged

2017	Mild depressive symptoms, along with persistent negative symptoms, had been observed	Sertraline 50 mg/day had been added to the treatment		Depressive symptoms had resolved, and negative symptoms had improved slightly

2017	Given the complexity of his clinical condition and the debilitating, fluctuating course of his symptoms, inpatient admission had been offered to facilitate close observation and optimization of treatment	After discharge, his treatment had consisted of zuclopenthixol decanoate 200 mg weekly, olanzapine 20 mg/day, and biperiden 4 mg/day		The greatest improvement observed to date had been achieved

2017–2021	Maintenance of outpatient follow‐up. Persistent negative symptoms were observed despite existing antipsychotic treatment. Subsequently, a bilateral hand tremor emerged	Fluoxetine 20 mg/day was added to target negative symptoms; propranolol 20 mg/day was initiated to manage the hand tremor		The patient’s tremor improved, and negative symptoms showed partial improvement

2021	Involuntary orofacial movements had emerged and, over the following months, had spread to the torso and extremities. The movements had been choreoathetoid in nature and had been alleviated with voluntary movement. No abnormal movements had been observed during sleep. The Abnormal Involuntary Movement Scale (AIMS) had been administered, and the total score had been 25. Neurological examination and neuroimaging had been unremarkable. A diagnosis of tardive dyskinesia had been made	Zuclopenthixol decanoate 200 mg weekly had been gradually discontinued, and olanzapine had been increased from 20 to 30 mg/day		The AIMS score had decreased to 20 in the following month; however, there had been an exacerbation of positive psychotic symptoms, including persecutory delusions

2021	VMAT2 inhibitors had been considered; however, his employers had canceled his insurance due to his limited attendance at the workplace. He had continued taking his antipsychotic medications with family support, and the family had declined to obtain VMAT2 inhibitors from abroad	Due to exacerbation of psychotic symptoms, amisulpride 400 mg/day had been initiated and had subsequently been increased to 1200 mg/day over the following months due to the severity of positive symptoms. Final treatment regimen had consisted of amisulpride 1200 mg/day, olanzapine 30 mg/day, fluoxetine 20 mg/day, propranolol 20 mg/day, and biperiden 4 mg/day		The AIMS score had remained around 20 with a fluctuating course. Positive symptoms had improved

2025	The patient was evaluated by the authors. He was still not attending work. Persecutory delusions, auditory hallucinations, Schneiderian first rank symptoms, and disorganized speech, thought, and behavior were prominent features of his clinical presentation. The PANSS total score was 171, and the AIMS score was 20. An electrocardiogram (ECG) was obtained, revealing a QTc of 420 ms	Lurasidone 20 mg/day was added to the treatment, with no changes made to the existing regimen		During the following week, the patient stated that he might return to work to check the environment and to see what was happening there. He reported increased motivation and had begun showering twice weekly. The PANSS total score was 161, and the AIMS score remained 20

2025	The patient agreed to the dose increase, and he also requested an increase in the dose of the newly initiated medication	Lurasidone dose was increased to 40 mg/day. No change has occurred in other drug treatments		During the following week, the patient began attending work three to four times per week and remained for full shifts. He reported that he had started conversing with some colleagues, although he still did not trust them. He had begun using public transportation, despite persistent hallucinations during transit. His supervisor reported that the patient appeared markedly improved compared to his previous condition and could be considered for full‐time employment and also encouraged him to attend work more frequently. The PANSS total score had decreased to 148. Choreoathetoid movements involving the mouth, trunk, and extremities had markedly diminished. The AIMS score was reevaluated and found to be 9

2025	A decrease in tardive dyskinesia symptomatology and negative symptoms has been observed	The dose of lurasidone was increased to 60 mg/day. In the following weeks, amisulpride was planned to be gradually discontinued, along with tapering of biperiden, and the dose of lurasidone was planned to be increased to at least 80 mg/day		No change in symptomatology was observed. The AIMS score was 9, and the PANSS total score was 148

2025	No change in symptomatology was observed. He continued to attend work and demonstrated improved self‐care and personal hygiene	The dose of lurasidone was increased to 80 mg/day, while amisulpride was reduced to 800 mg/day, and biperiden was reduced to 2 mg/day		No change in symptomatology was observed. The AIMS score remained 9, and the PANSS total score was 148

2025	No change in symptomatology was observed	The dose of lurasidone was maintained unchanged, while amisulpride was reduced to 400 mg/day, and biperiden was discontinued		No emergence of parkinsonism was observed. The AIMS score was 9, and the PANSS total score was 148. Positive symptoms remained stable; however, the patient continued to exhibit delusional beliefs

2025	No change in symptomatology was observed	Amisulpride was discontinued. The final treatment regimen consisted of lurasidone 80 mg/day, olanzapine 30 mg/day, fluoxetine 20 mg/day, and propranolol 20 mg/day		The PANSS total score was 148, and the AIMS score was 9

*Note:* QTc: corrected QT interval; mg/day: milligrams per day; mg/week: milligrams per week; mg/monthly: milligrams per month.

Abbreviations: AIMS, Abnormal Involuntary Movement Scale; ECG, electrocardiogram; PANSS, Positive and Negative Syndrome Scale.

**Table 2:** Changes in clinical scale scores during lurasidone treatment and clinical evaluation of the patient.

Clinical scales and parameters	2021 (first TD evaluation)	Baseline—week 0 (before lurasidone)	Week 2 (lurasidone 20 mg/day)	Week 3 (lurasidone 40 mg/day)	Follow‐up (lurasidone 60–80 mg/day)
PANSS scores					
Positive scale	N/A	43	42	40	40
Negative scale	N/A	42	39	33	33
General psychopathology	N/A	86	80	75	75
Total PANSS score	N/A	171	161	148	148
AIMS scores					
AIMS facial and oral movements	15	12	12	7	7
AIMS extremity movements	6	4	4	0	0
AIMS trunk movements	4	4	4	2	2
AIMS total	25	20	20	9	9
AIMS global judgement	11	8	4	4	4
AIMS dental status	No	No	No	No	No
AIMS sleep disappearance	Yes	Yes	Yes	Yes	Yes
CGI‐S	—	6	5	4	4
CGI‐I	—	4	3	2	2
Clinical evaluations					
Lab parameters	CBC: normal physiological limits; ferritin: 262 µg/L; eGFR: 74.44 mL/min/1.73 m^2^; CPK: 99 U/L; Na^+^: 140 mmol/L; K^+^: 4.78 mmol/L; Ca^+2^: 9.36 mg/dL; CRP: 0.59 mg/L; AST: 15.5 U/L; ALT: 17.9 U/L; LDH: 266 U/L				
ECG (QTc, ms)	—	420 ms	—	—	—
MRI report	The supratentorial and infratentorial brain parenchyma demonstrate normal morphology and signal characteristics. No evidence of mass effect or herniation is observed. The ventricular system is normal in size and configuration. Cortical sulci and convexity are appropriate for the patient’s age. The cerebellum, brainstem, and the seventh and eighth cranial nerves are normal. There is no diffusion restriction on diffusion‐weighted imaging (DWI)				
Neurological evaluation	The patient was alert, oriented, and cooperative. Deep tendon reflexes were symmetric and within normal limits. Motor strength was 5/5 in all muscle groups. No upper or lower motor neuron signs were observed. Cerebellar examination was unremarkable. Cranial nerves were intact. Gait and coordination were normal. No extrapyramidal signs, including cogwheel rigidity, were detected, and there was no tremor or abnormal posturing. However, choreoathetoid movements were observed in the extremities, torso, and orofacial region				

*Note:* Na+, sodium; K+, potassium; Ca + 2, calcium; mg/day, milligrams per day; QTc, corrected QT interval.

Abbreviations: AIMS, Abnormal Involuntary Movement Scale; ALT, alanine aminotransferase; AST, aspartate aminotransferase; CBC, complete blood count; CGI, Clinical Global Impression; CGI‐I, Clinical Global Impression–Improvement; CGI‐S, Clinical Global Impression–Severity; CPK, creatine phosphokinase; CRP, C‐reactive protein; ECG, electrocardiogram; eGFR, estimated glomerular filtration rate; LDH, lactate dehydrogenase; MRI, magnetic resonance imaging; N/A, not assessed; PANSS, Positive and Negative Syndrome Scale; TD, tardive dyskinesia.

**Table 3:** Differential diagnosis of other conditions and clinical reasons for exclusion.

Differential diagnosis	Reason to exclude
Withdrawal emergent dyskinesia	Tardive dyskinesia of the patient had been developed under zuclopenthixol decanoate treatment, and at the time, tardive dyskinesia had been developed, and there had been no antipsychotic drug dose decrease
Acute dyskinesia	The patient had developed dyskinesia symptoms over months, a year roughly. During this time period, there had been no treatment change or there had been no dopamine agonist medication that the patient had been receiving
Stereotypy	AIMS evaluation had/has been conducted according to the scale manual in which the patient’s movement was evaluated while sitting in a chair at a resting position, hands were on the knees, and feet were flat. Also, the patient’s hands were asked to hang between legs, and again he was asked to protrude his tongue. He was examined while his arms were extended one at a time. Also, he was asked to tap his thumb during facial evaluation. His arms were examined, while both were outstretched. Finally, his gait and torso were examined while both at rest and while he was walking. Also, the patient was aware of his movements, and he was disturbed by them
Drug‐induced parkinsonian tremor plus oral movement	The patient’s movements were choreoathetoid with low frequency, and his movements were not usually repetitive. The patient’s hands were twitching and bending rather than repetitive shake or like a parkinsonian tremor. His oral movements were also frequently changed in frequency with gross rolling movement without jaw tremor. His symptoms were absent during sleep. Cogwheel rigidity was absent
Functional movement disorder	The patient reported being distressed by his dyskinetic movements throughout the evaluation. The severity of symptoms remained stable and was not influenced by psychosocial factors or variations in affective state
Spontaneous dyskinesia	The patient’s dyskinesia symptoms had occurred while he had been at relatively better symptomatology than previous conditions. Also, his dyskinesia had been alleviated little, while zuclopenthixol deconate had been gradually reduced; however, his positive symptoms had been increased in severity. Due to this discordance of symptomatology and dyskinesia, spontaneous dyskinesia had been excluded. Moreover, his thought content was extremely occupied by delusions even his severity of dyskinesia was decreased

Abbreviation: AIMS, Abnormal Involuntary Movement Scale.

## 3. Discussion

In this report, we describe an improvement in TD following the addition of lurasidone to the treatment regimen in a patient with treatment‐resistant schizophrenia who had previously been treated with olanzapine and amisulpride. Our patient had developed TD in 2021, with an AIMS score of 25, and the condition persisted until lurasidone therapy was added in 2025. In addition, the patient had experienced severe functional impairment due to persistent psychotic symptoms. After the initiation of lurasidone, he began engaging in self‐care activities such as showering and was able to return to work, even though positive psychotic symptoms largely persisted. A meaningful reduction in negative symptomatology was observed even though the real‐world data suggest that lurasidone doses of at least 80 mg/day are effective in reducing the PANSS score in treatment‐resistant schizophrenia. The observed improvement may be partly explained by polypharmacy that our patient has been using [14].

Regarding the etiology of TD, the earliest hypothesis was the dopaminergic hyperactivity hypothesis, which stemmed from chronic D_2_ receptor blockade. However, postmortem studies did not consistently confirm this hypothesis, necessitating alternative models. These include the D_3_ receptor hyperactivity hypothesis and oxidative stress caused by increased dopamine turnover in the brain. Genome‐wide association studies have suggested that genes related to D_2_R, D_3_R, 5‐HT_2_A, and 5‐HT_2_C receptors, as well as genes involved in oxidative stress pathways, may contribute to the development of TD [15].

In the current literature, there are reports indicating that lurasidone may induce TD or other tardive syndromes [10–13]. Tripathi et al. highlighted the relatively high dopaminergic affinity of lurasidone (Ki = 0.994 nM) [13]. Our patient was also receiving amisulpride, which has a lower D_2_ receptor binding affinity than lurasidone (~30 nM) [16]. Olanzapine is also known to have relatively low D_2_ affinity among atypical antipsychotics. The improvement in TD observed in this case cannot be readily explained by a simple switch to an antipsychotic with lower D_2_ potency. In addition, TD is associated not only with the potency of antipsychotics but also with D_2_ receptor occupancy of the current treatment; therefore, reduction of the antipsychotic dose may alleviate the symptoms of TD in some cases [17]. However, in our case, the patient had been receiving amisulpride and olanzapine prior to the initiation of lurasidone, and the doses of these medications remained unchanged until symptom improvement was observed. Another contributing factor to TD is polypharmacy and the use of anticholinergic agents. In our case, the polypharmacy burden initially increased rather than decreased, and the dose of biperiden remained stable during the period in which improvement in movements was observed. Therefore, it cannot be definitively concluded that lurasidone directly treated TD in this case, but receptor interactions involving lurasidone, amisulpride, and olanzapine, as well as biperiden and propranolol, may have contributed to this improvement. Nevertheless, the major confounding factors remained stable during the period of amelioration of TD.

Clozapine is known to alleviate symptoms of TD [18], and Ishibashi et al. reported several pharmacological similarities between clozapine and lurasidone [8]. One such similarity is the comparable relative potency at 5‐HT_1_A, 5‐HT_2_A, and 5‐HT_7_ receptors. The affinity of both clozapine and lurasidone for these receptors is hypothesized to contribute to their effects on TD. Notably, the 5‐HT_2_A receptor gene has been identified as a susceptibility gene for TD [19], and amelioration of symptoms is further hypothesized to be related to 5‐HT_2_A receptor blockade by lurasidone. Another similarity with clozapine noted by Ishibashi et al. is that lurasidone increases dopamine turnover preferentially in the prefrontal cortex rather than in the striatum [8]. Increased striatal dopamine turnover and the resulting oxidative damage have been proposed as one of the pathogenic mechanisms underlying TD even though the results of trials with antioxidants are conflicting and a direct association could not be established [15]. Within the scope of lurasidone, there is only preclinical evidence suggesting that the drug may exhibit antioxidant activity [20, 21]. Therefore, preferential effects in the prefrontal cortex and 5‐HT_2_A receptor blockade are hypothesized to contribute to the observed improvement in TD symptoms.

In cases where clozapine treatment cannot be utilized, polypharmacy may be an option, and there are examples in the literature [22]. Several studies have reported that lurasidone augmentation is associated with reductions in PANSS scores in treatment‐resistant schizophrenia [23–25]. Consistent with these findings, our patient experienced a reduction in negative symptoms and was able to resume working, which may reflect increased social engagement, as also reported in the cohort studied by Maruyama et al. [26].

## 4. Limitations

This patient had a long history of treatment‐resistant psychotic symptoms, during which multiple antipsychotics had been trialed. Combination treatment had been required because of persistent functional impairment and psychotic symptoms. In addition, the patient could not afford VMAT2 inhibitors, which represent the first‐line treatment for TD, and clozapine treatment could not be initiated due to concerns regarding adherence and severe psychotic symptoms. Also, biperiden and amisulpride were continued for a period of time. Both may also have aggravated the patient’s TD.

## 5. Conclusion

In this case, lurasidone is hypothesized to improve TD and negative symptoms in a patient with treatment‐resistant schizophrenia. The mechanism underlying this effect might stem from various different mechanisms including different receptor interactions. Further studies are required to clarify the underlying mechanisms, including investigations using genotyping and neuroimaging.

## Author Contributions

Umut Günay evaluated the patient and started the treatment. Elif Tatlıdil reevaluated the patient. Umut Günay, Candost Demiröz, Fatma Seher Kocaayan, and İlay Dalkıran followed the patient and scored the particular scales. Umut Günay, Candost Demiröz, Fatma Seher Kocaayan, and İlay Dalkıran prepared the first draft of the case report. Elif Tatlıdil corrected the draft.

## Funding

This study received no specific funding from any public, commercial, or not‐for‐profit sectors.

## Disclosure

ChatGPT 5.2 was used only for grammar correction of this case report.

## Ethics Statement

Institutional Review Board approval was not required for this single anonymized case report.

## Consent

Written informed consent was obtained from the patient and can be provided upon request. Personal information was not included in the case report in order to protect the patient’s anonymity.

## Conflicts of Interest

The authors declare no conflicts of interest.

## Data Availability

The data that support the findings of this study are available from the corresponding author upon reasonable request.
